# The experiences of early postpartum Shenzhen mothers and their need for home visit services: a qualitative exploratory study

**DOI:** 10.1186/s12884-019-2686-8

**Published:** 2019-12-31

**Authors:** Xiao Xiao, Fei-wan Ngai, She-ning Zhu, Alice Yuen Loke

**Affiliations:** 1 School of Nursing, The Hong Kong Polytechnic University, GH 525, Hung Hom, Kowloon, Hong Kong; 2 Department of Obstetrics, Affiliated Shenzhen Maternity & Child Healthcare Hospital, Southern Medical University, Shenzhen, China; 3 Department of Nursing Administration, Affiliated Shenzhen Maternity & Child Healthcare Hospital, Southern Medical University, Shenzhen, China

**Keywords:** Women’s needs, Postpartum care, Social support, Transition to parenthood / grandparenthood, Intergenerational conflicts

## Abstract

**Background:**

The early postpartum period is the most stressful period for a new mother, who is assuming new roles and responsibilities in life, and must deal with the demands from her newborn baby and her own care needs. Little is known about whether the current postnatal care services provided by hospitals and community centers meet the needs of women. The aim of this study was to identify the experiences of women in Shenzhen and the problems that they encountered during the first 6 weeks after giving birth; and to explore their expressed needs with regard to postnatal care services.

**Methods:**

This is a qualitative exploratory study. Data were collected in November 2018 through in-depth, semi-structured, face-to-face interviews. A purposive sample was recruited from a tertiary maternal hospital in Shenzhen, China. The dataset was analyzed using content analysis.

**Results:**

Twenty-two mothers were interviewed during their postpartum body check on the 30th or 42nd day after giving birth. Six themes were identified: “the self-care needs of women,” “proficiency in infant care,” “involvement of family in postpartum and infant care,” “family conflicts over postpartum and infant care,” “preparing for the transition to parenthood / grandparenthood,” and “the need for comprehensive postpartum home visit services.”

**Conclusions:**

The concerns expressed by the women during the postpartum period were related to their need to recover physically and to their desire to be perceived as proficient in infant care. Support from husbands and grandmothers could facilitate or impede a woman’s transition to motherhood, and the family’s transition to parenthood / grandparenthood. There were disagreements arising from intergenerational beliefs about postpartum and child care. In providing postpartum care services to women in situations where the family is involved in their care, health professionals should consider the family as a whole.

## Background

The postpartum period is both a happy and crucial period for a woman and her entire family. The early postpartum days are the most stressful period for a mother, who must deal with demands from her newborn baby and her own care needs [1], while coping with physiological and psychological changes [2]. In a survey conducted in the United States, about 42% of women were found to suffer from physical and psychological distress because of their perceived inability to care for their newborn [3]. Struggling with the demands of caring for an infant, alterations in body image, and changes in family composition are also possible stressors during the postpartum period [4]. New mothers need support from their family in the first 6 weeks after giving birth [5]. Support from family members can enhance a woman’s self-efficacy, confirm her role and identity as a mother [6], and improve her general health and social well-being, and the health of the newborn child and family [4].

The guidelines on postnatal care for mothers and newborns from World Health Organization (WHO) highlighted the significance of postpartum home visits to optimize their health outcomes. The guidelines recommended that physical assessment of the newborns and mothers, counselling, and psychosocial support should be included in the contents of home visits [7]. The effectiveness of postpartum home visits have also been confirmed. For example, two randomized controlled trials in western countries compared the differences between home-based postpartum care (early discharge) and hospital-based care, they found that home-based postpartum care is safe and effective with regard to physical parameters, breastfeeding and postpartum depression for low risk pregnancies [8, 9].

In China, the government stipulates that nurses are to visit the homes of all postpartum women. However, due to a lack of standardized guidelines for postpartum home visits and the lack of specific training for nurses, the home visit services have been criticized for not meeting the needs of women [10]. The overall usage of postpartum home visits and the level of satisfaction with the services are reportedly quite low, and do not meet the needs of postpartum women and their families [11]. A study in Tianjin reported that the postpartum home visit services are not comprehensive, and that women wanted the services to include the teaching of postpartum self-care, support for breastfeeding, and contraception [12]. A study carried out in Beijing revealed that about 28.4% of postpartum women preferred to hire a maid when “doing the month” (a lying-in period following birth according to the Chinese tradition) and refused home visit services by nurses [13]. Another study conducted in Guangzhou reported that only 57% of women accepted the offer of postpartum home visit services [14]. These studies suggest that, while well intentioned, home visit services have failed to meet the needs of postpartum women [13, 14].

A document titled “Guidance for the transformation and development of nursing services” was released in 2018 by the Chinese Ministry of Health together with 11 other relevant ministries as a call to action [15]. Emphasized in the document was the importance of promoting quality maternal and infant care as a strategic initiative [15]. In complying with this call from the government, clinicians and scholars have begun to explore ongoing, quality, and effective postnatal nursing care in the community. Many related studies have been conducted, including several cross-sectional studies on the needs of postpartum women, such as for contraception or breastfeeding advice [13, 14]. However, these studies were designed based on the ‘nominative needs’ of health professionals, without addressing the expressed needs of postpartum women. Thus, this study was launched to gain a better understanding of the needs of these women, in order to develop appropriate services for them and their families to enhance the health and well-being of the women and their newborn infants.

## Methods

### Maternal and child healthcare services in Shenzhen

Shenzhen is a major metropolis in Guangdong Province, China, which lies north of the border with Hong Kong. The population numbered 11.9 million in 2016. On average, Shenzhen residents are only 32.5 years old, and 67.7% are migrants from other parts of China who have come in pursuit of better job opportunities and living conditions [16].

There are three layers of maternal and child healthcare services in Shenzhen. The Shenzhen Maternity and Child Healthcare Hospital is the only tertiary level major maternity hospital in the area and is the referral center for all high-risk pregnancies. The second layer is comprised of ten municipal maternity and child healthcare hospitals – one in each district in Shenzhen. The third layer is made up of community health service centers under the municipal hospitals, which provide services to residents in the community. According to the Shenzhen Maternal and Child Health Statistics Department (2017), the number of infants delivered in Shenzhen in 2016 was 212,818, around 20,000 of whom were delivered in the Shenzhen Maternity and Child Healthcare Hospital.

#### Postpartum services of the Shenzhen maternity and child healthcare hospital

The Shenzhen Maternity and Child Healthcare Hospital (SMCHH) was the first to initiate home visit service programs for postpartum women in Shenzhen back in 1999 [17]. The take-up rate for such programs in Shenzhen is 97.4%, much higher than the national rate of 85% [18, 19]. The program provides for a first home visit by a nurse within two days after a woman is discharged from the postnatal unit, with follow-up visits being handled by the community health centers. A home visit nurse monitors a mother’s physical recovery and removes stitches if needed; monitors the development of the newborn and conducts a blood test to screen for genetic diseases; and gives new mothers instructions on umbilical cord care, the feeding of babies, and the intake of nutrients for both new mothers and newborns. Visiting nurses will also remind women to return to the hospital between the 30th and 42nd day after the delivery, to undergo a postpartum body checkup at the women’s health center and for the baby to undergo an assessment at the child healthcare center of the same hospital. According to statistics from the SMCHH, around 90% of postpartum women return for a physical checkup and child assessment.

Although the SMCH hospital has been offering its postpartum home visit program for nearly 20 years, the service has not been evaluated. Little is known about whether the services provided by the hospital and the community centers meet the needs of the women, and whether other services should be included. This is a qualitative study to explore the experiences and service needs of postpartum women in Shenzhen.

### Research aims and objectives

The aim of this qualitative study is to explore the concerns and well-being of women during the early postpartum period, and to identify their health service needs. The findings of this study will provide directions for developing the home visit program, and possible interventions for improving the postnatal care provided to women in Shenzhen, to assist them in making a smoother transition towards motherhood.

The objectives of this study were:

1. To explore the physical, psychological, and social concerns of Shenzhen women in the first 6 weeks after giving birth.
2. To identify the experience and difficulties encountered by Shenzhen women in the first 6 weeks after giving birth.
3. To explore the expressed service needs of Shenzhen women in the first 6 weeks after giving birth.

### Study design

This study was a qualitative exploratory study. An understanding of the experiences and health needs of individuals should be explored in the context in which they are embedded [20]. Because of the researcher’s experience of living and working with postpartum women in Shenzhen, the results of the study are interpreted in the context of the situation in Shenzhen.

#### Target participants

First-time mothers are said to be less experienced in dealing with the difficulties encountered during the early postpartum period and to have more demands for supportive care than experienced mothers [5]. However, with the recent announcement of the “second child” policy in China in 2016, the birth of a second child could give rise to other concerns for women in China relating to family dynamics and relationships [21].

Both first-time and second-time mothers were recruited using a purposive sampling strategy. Women who gave birth at the SMCHH and returned to the women’s health center for a checkup on the 30th or 42nd day following the delivery were invited to participate. Women were recruited for interviews in accordance with the criteria for inclusion and exclusion. Recruitment continued until data saturation was reached, when no new information emerged from the data [22].

##### Inclusion and exclusion criteria

Women were included if they: (1) had given birth to a healthy baby in the last 6 weeks in the SMCHH; (2) were a first or second-time mother; (3) without pregnancy or postpartum complications; (4) had delivered a singleton baby at full term between 37 to 42 weeks, with a birth weight of ≥2.5 kg; and (5) were living in Shenzhen in the same household as their husband. Women who (1) had gestational diabetes, hypertensive disorders, or any other complications; (2) could not read or speak Mandarin; (3) reported having mental health problems; (4) had a known chronic disease; and (5) whose baby had been admitted to the Neonatal Intensive Care Unit or had abnormalities were excluded.

### Data collection and procedure

#### Recruitment

Permission was sought from the head nurse of the women’s health center to approach women who were returning for a postpartum checkup on the 30th or 42nd day after giving birth. The medical records of the women were checked for eligibility. Those who were eligible were approached and given an explanation of the purpose of this study, and were invited to take part in it. Those who consented to take part were interviewed in a private room of the department. Since most of the women were accompanied by a family member, their baby was cared by the family member during the interview.

#### Data collection

Semi-structured face-to-face interviews were conducted in November 2018 by the researcher, who is a registered midwife in the hospital. Before the interview, the women were encouraged to cater to the feeding or other needs of the baby, so that they would be more relaxed during the interview and less likely to be distracted. The interviews were conducted in a quiet room away from disturbances, to give the participants the comfort to freely express their concerns.

The demographic information of the women, the infant, and the family caregivers were collected. The demographic information sheet (Additional file 1) and the interview guide (Additional file 2) were delineated based on the researcher’s experience in working with postpartum women, and discussed with two academics in obstetric nursing and family nursing. Each interview lasted for about 25 to 45 min. The interviews were audio-taped and field notes were taken. Those who completed the interview were offered small items for the baby in appreciation, such as diapers. As no new information was revealed by the women after the 20th interview, two more interviews (21st and 22nd) were conducted before the interviewing process was brought to a close, as data saturation had been reached.

#### Interview technique

The researcher is an experienced maternity nurse who works in the same hospital and has experience in conducting interviews. At the beginning of the interview, the researcher introduced herself to the women to establish rapport. The interview began with the question “Please tell me about your concerns / experiences in this postpartum period.” Questions were asked to solicit further information where appropriate. The women were also asked for their opinions about the home visits, and whether they had any suggestions for improvement and additional services. At the end of the interview, to obtain information that might be relevant the following open-ended question was asked: “Is there anything else you would like to tell me?”. The women were encouraged to freely express their views.

### Data analysis

Content analysis was employed to analyze the interview data [23]. Field notes were also taken and analyzed [20]. The researcher transcribed the audio-taped interviews within a week of conducting the interviews, and the two other researchers checked the accuracy of the transcripts.

The NVivo 11 software was utilized to help with analyzing the data, specifically, in identifying common codes from the transcripts. The coding scheme and identified themes were then discussed among the three researchers in the team. A bracketing strategy was used during the process of analyzing the data to reduce the problem of bias. A summary of the demographic information of participants is given in Table 1.

**Table 1:** Demographics of postpartum women (*n* = 22)

Demographic	Number (%)
Age	
< 25	1 (4.5%)
25–34	17 (77.3%)
> 34	4 (18.2%)
Parity	
Primipara	14 (63.6%)
Multipara	8 (36.4%)
Mode of delivery	
Vaginal Delivery	12 (54.5%)
Assisted Vaginal Delivery	1 (4.5%)
Planned Cesarean Section	6 (27.3%)
Emergency Cesarean Section	3 (13.6%)
Gender of the newborn	
Girl	13 (59.1%)
Boy	9 (40.9%)
Type of Feeding	
Exclusive breastfeeding	13 (59.1%)
Mixed feeding	9 (40.9%)
Maternal Leave (days)	
60–120	1 (4.5%)
121–180	12 (54.5%)
181–240	5 (22.7%)
Not applicable	4 (18.2%)
Caregiver in postpartum period	
Mother-in-law	7 (31.8%)
Husband	1 (4.5%)
“Doing the month maid”	1 (4.5%)
Mother-in-law and mother	4 (18.2%)
Mother and “doing the month maid”	6 (27.3%)
Mother-in-law and “doing the month maid”	3 (13.6%)
Hometown of caregiver	
Urban	10 (45.5%)
Suburban	12 (54.5%)
Monthly household income (RMB)	
5000–10,000 RMB	7 (31.8%)
10,001-20000RMB	7 (31.8%)
20,001-30000RMB	5 (22.7%)
> 30001RMB	3 (13.6%)

### Ethical considerations

The aim of the study was explained to the participants. They were interviewed on a voluntary basis and could withdraw from the study at any time. The women had the freedom to choose to take part in or refuse to participate in this study, and would be treated equally at the health center regardless of their decision. The participants were given assurances that their confidentiality would be maintained. The questionnaires and transcripts of the interviews were coded with case numbers that did not reveal the identities of the participants. The audio-tapes were available only to the research team and kept in a locked cabinet. At the end of the study, the audio-tapes were destroyed.

### Methodological rigor

The participants were recruited strictly according to the eligibility criteria. When it seemed that the point of data saturation had been reached, the researcher continued to interview two more women to verify that this was the case [22]. Two researchers read the transcripts and codes independently. They then held a meeting to discuss the codes and themes that had been identified, and read the results of the study to cross-check the interpretations for accuracy. If they could not reach a consensus, the third researcher was included for a further discussion. The above strategies were adopted to enhance the trustworthiness of the findings.

## Results

### Demographic information of the participants

A total of 22 participants were recruited and interviewed. The women were between 22 to 43 years of age (mean = 31.75 ± 4.35 years old). Among them, 14 were first-time mothers and 8 were second-time mothers. They had lived in Shenzhen for 1 to 29 years (mean = 11.65 ± 6.68 years). More than half of the women (*n* = 12, 54.5%) reported having a monthly household income of between 10,001 to 30,000 RMB (US$1455 - $4366).

Thirteen out of the 22 (59.1%) women had a vaginal delivery. The women were taking an average of 183.33 ± 27.49 days of maternity leave. All of the women continued to breastfeed, with 9 (40.9%) breastfeeding exclusively and 13 (59.1%) breastfeeding partially at the 42nd day after giving birth. During the “doing the month” early postpartum period, which is a Chinese tradition, 4 (18.2%) were cared for by both their mother and their mother in-law, 10 (45.5%) by their mother-in-law with or without the aid of a “doing the month maid,” and 6 (27.3%) by their mother together with a “doing the month maid.” One woman hired a “doing the month maid” and one was cared for mainly by her husband. Of the mothers / mothers-in-law, 12 (54.5%) came from suburban areas of China, and 10 (45.5%) from urban areas (Tables 1).

Six main themes emerged from the analysis of the interview transcriptions. They were: “the self-care needs of women,” “proficiency in infant care,” “involvement of family in postpartum and infant care,” “family conflicts over postpartum and infant care,” “preparing for the transition to parenthood / grandparenthood,” and “the need for comprehensive postpartum home visit services” (Table 2).

**Table 2:** Summary of themes and categories from the findings

Themes	Categories
Self-care needs of women	Physical discomfort and recovery
	Need for nutritional supplements
Proficiency in infant care	Baby’s feeding
	Baby’s elimination
	Baby’s skin rashes
	Baby’s crying
Involvement of family in postpartum and infant care	Father’s support in child care
	Support from grandmothers in child care
Family conflicts over postpartum and infant care	Disagreement over nutritional supplements for postpartum women
	Conflicts over infant care practices
	Arguments or cold wars in multigenerational families
Preparing for the transition to parenthood / grandparenthood	Making arrangements before the arrival of the newborn
	Offer husbands advice on supporting new mothers
	Offer grandparents support in baby care
	Facilitate intergenerational communication
The need for coordinated and comprehensive postpartum home visit services	Appreciate the attention paid by home visit nurses to the psychological well-being of new mothers
	Request for an online support program
	Provide jaundice testing at home
	Provide support for breastfeeding in home visits

#### The self-care needs of women

The women who were interviewed were concerned about their physical discomfort and recovery, and about the need to take nutritional supplements in the early postpartum period. While the first-time mothers were concerned about changes in their body, the second-time mothers believed that their physical health was worse than it had been after their first delivery.

### Physical discomfort and recovery

In this early postpartum period, most women were concerned about the various forms of physical discomfort that they were experiencing after childbirth. These involved pain from wounds from a cesarean section / perineal tears, nipple soreness from breastfeeding, and injuries to their waist and wrists. Women were also concerned about the loss of elasticity in their pelvic floor muscles.

Due to perineal tears during delivery, women experienced indescribable feelings and pain. They wanted to know if their vaginal area was healing properly, and how to determine whether their lochia was normal. They were embarrassed to ask, but anxious about the physical changes that they were experiencing and about the healing process. They wished that someone would tell them how they were doing.

*I am curious about my body, particularly my perineal healing and lochia discharges. I learned that it is normal for it to take as long as 30–40 days, but I am still nervous about some bloody lochia that I have discharged.* (P19 – first-time mother).

They complained about the discomforts caused by childbirth, with pain coming from a cesarean wound or a perineal tear from a vaginal delivery.

*I have discomfort from the cesarean wound on my tummy. When I bend over to wash my baby, it causes pain. I feel that I cannot stand straight at the waist / tummy after I had the cesarean section.* (P6 – second-time mother).

While women enjoyed breastfeeding their baby, which gave them a wonderful feeling of motherhood, they also complained of sore nipples. Some women had cracked nipples and felt pain when breastfeeding, but felt too guilty to stop breastfeeding their baby. Women wanted to learn more about caring for their nipples.

*Somehow, each time I feed the baby, she could not snatch on the nipples very well, and I felt pain. The skin on my nipples is breaking and bleeding. I am scared every time the baby sucks on my nipples.* (P17 – first-time mother).

*My baby boy is strong. He sucks intensely and powerfully, and both of my nipples are sore* …. (P3 – first-time mother).

*I had galactostasis, then acute mastitis. My inflamed breasts are painful … it’s torture. I will have to stop breastfeeding.* (P21 – first-time mother).

Caring for and holding the baby also caused injuries to the waist and wrists of the new mothers. Women reported having waist and wrist pain from bending over and holding the baby when changing diapers or breastfeeding. As the baby got heavier, the pain worsened.

*I have pain in my wrists. It is stiff and painful when I turn my hands. It is worse when I have to hold the baby in my arms, and hold my breast for feeding.* (P3 – first-time mother).

*She cries a lot, so I have to hold her in my arms. My waist is sore, and I hope I can get some rest if somebody else can hold her for a while.* (P6 – second-time mother).

Some women worried about a loss of elasticity in their pelvic floor muscles, leading to urination incontinence. They had embarrassing moments when they felt an urgent need to urinate or experienced leakages when they sneezed. They heard stories about how pelvic floor muscles can be damaged during a vaginal birth, and now their urinary incontinence worried them. They wanted more information on whether their muscles would recover elasticity.

*My pelvic floor muscles have not regained strength after delivery. I experience urgency and frequency of urination. I also experience leaks when I laugh. I am not sure if the muscles need to be repaired. My friend told me that she has been incontinent since giving birth a few years ago. I want to know if the injury to my pelvic floor is serious …*. (P4 – first-time mother).

Besides the issues of physical discomfort and recovery, the women also described themselves as being “weak” from sweating. In Chinese, “sweat” is “xu,” which is similar to the English term for “weak.” The women believed that the process of giving birth made them weak in that they lost energy (“qi” in Chinese) in their body. They lost bodily strength from the exhausting process of going into labor, and also lost body fluids, experienced bleeding, wounds (breaks in their skin, which allows “qi” to escape), and pain. So they needed to recuperate to regain their strength, to meet the demands of caring for a baby.

*I need to have a longer postpartum “doing-the-month” period to have a good rest. I need to recover and regain my strength so I can have the energy to take care of my baby …*. (P11 – second-time mother).

### Need for nutritional supplements

According to the Chinese culture, nutritional supplements are important in the postpartum recovery process. Women are considered “xu” (weak) after giving birth and are thought to require nutritional supplements to regain their strength, in order to balance the “ins” and “outs.” The belief is that if the “outs” from childbirth are not supplemented with appropriate and sufficient “ins” after delivery, women will not recover their pre-pregnancy state. The women expressed a need for nutritional supplements for a better recovery.

*I did not like the food prepared by my mother. If economic conditions had permitted, I would have wanted to hire a “doing the month maid” to assist me by preparing nutritious and tasty “postpartum meals.” A “doing the month maid” will be more professional than my mother in cooking “postpartum meals” that will bring about a faster recovery.* (P6 – second-time mother).

Apart from their need for nutrients to achieve a better recovery, the women believed that their physical health would affect the development of their baby.

*I would like to eat more nutritious foods to produce better-quality breast milk for my baby.* (P5 – first-time mother).

*I want to have tonic foods to regain energy and avoid any comorbidities. Otherwise, I would not be able to take good care of my baby and provide her with nutritious breast milk.* (P11 – second-time mother).

#### Proficiency in infant care

Surprisingly, not only the first-time mothers, but also the second-time mothers were concerned about their proficiency in caring for their infant. They were concerned about the baby’s feeding, elimination, skin rashes, and crying. The mothers were anxious about everything related to caring for their newborn baby.

### Baby’s feeding

Mothers were concerned that they were not producing enough breast milk for their baby. Some would supplement their feeding with baby formula. In not knowing how to assess whether the baby was consuming enough, many used the baby’s weight gain as a reference.

*My baby weighed 3.45 kg at birth, and she weighed only 100 g more on the 15th day. My sister-in-law suspects that I do not produce enough milk and said that I should feed my baby with formula. So I added formula to my feeding.* (P4 – first-time mother).

### Baby’s elimination

Besides feeding, the mothers also scrutinized their baby’s stool to determine the baby’s well-being. The women usually did not know what to look for, and were frightened at the appearance of the baby’s stool.

*At the first few days after birth, I thought the baby was having diarrhea. I then became nervous. Then, there were times when, with his red face, he looked like he was straining to eliminate stool; I would get anxious again*. (P12 – first-time mother).

### Baby’s skin rashes

It was a nightmare for mothers to see skin rashes on a fragile baby. Women were frustrated with rashes that did not heal.

*My baby has skin rashes that do not heal, and I was told he has eczema. I don’t know how to deal with it, but was told not to put ointment. It must be painful, and it hurt me to see the rashes. There are times when it gets a bit better, but I worry about recurrences.* (P11 – second-time mother).

### Baby’s crying

Babies cry when they are hungry, if their diaper is wet, or if they do not feel well. The women were confused about the crying, and became frustrated when they could not comfort the baby. When there was no way to stop the baby from crying, they would calm the baby by holding the baby in their arms.

*I am very nervous when my baby cries; I don’t know what is really wrong with him. Then I begin to worry and feel like a fool.* (P12 – first-time mother).

*I would read books or surf the internet to find information on a baby’s cries. I wanted to know how to differentiate between the cries.* (P20 – first-time mother).

*I stayed up for three days …; she would fall asleep when she was sucking, then if I put her to bed she would start crying again. I would pick her up and hold her in my arms until she fell sleep again. I kept doing this all the time, until daylight.* (P14 – second-time mother).

#### Involvement of the family in postpartum and infant care

Women were desperate to receive support from their husband and extended family members in postpartum and infant care. It was notable that first-time mothers made more requests to family members for support. Such support meant a lot to the mothers. They hoped that family members would help them care for their child, rather than be a burden on them.

### Father’s support in child care

A few mothers complained that the baby’s father was not assuming any responsibility for caring for the baby. For the fathers, the baby was just like a doll to play with.

*My husband will play with the baby, but as soon as the baby cries, he will quickly hand the child over to me. I hope he realizes his role as a father and provides me with childcare support.* (P3 – first-time mother).

It was obvious that mothers were more satisfied if their husband shared the responsibility of caring for the baby. The attention and readiness of fathers to help meant a lot to the women. With their husband’s support, the women felt loved, supported, and protected.

*My husband is great. He takes care of the baby after he returns from work. Our baby laughs when he plays with him, which he does not do with me. When the baby is hungry at night, he will get up and heat up milk from the refrigerator. He is good at helping and I am grateful*. (P10 – first-time mother).

### Support from grandmothers in child care

In Chinese traditional culture, parents, as extended family members, are involved in caring for their grandchild. The mother / mother-in-law of the women are eager to provide help in caring for the new mother and baby. The women usually felt more comfortable with their own mother, who understood them and whom they found easier to talk to.

*Actually, I wanted to go to a “doing the month” maternity hotel, but my mother-in-law insisted that she would take care of me. Due to her traditional beliefs, she feels that she should help, since my husband is her only son. I would love to return to my parents’ home for postpartum support. It would be easier to talk to my own mother about my needs. My mother would be able to comfort me and provide me with psychological support.* (P10 – first-time mother).

Some women considered themselves lucky to have both mothers work in harmony when helping them. Each of the mothers took up different responsibilities to provide the needed postpartum and infant care.

*Both my mother and mother-in-law came to Shenzhen from their hometown after I gave birth to support me in “doing the month.” My mother cooks the nutritious food that I am used to. My mother-in-law does the cleaning or other necessary household chores, while I focus on caring for the baby.* (P14 – second-time mother).

#### Family conflicts over postpartum and infant care

While some women appreciated the support that they received from their husband and mother / mother-in-law, not all could work together in harmony due to different postnatal care beliefs and childcare practices in different provinces in China. Both the first-time mothers and second-time mothers were concerned about the “old ways” of their mother / mother-in-law in infant care. Some families had disputes arising from different expectations.

### Disagreements over nutritional supplements for postpartum women

Determining what a woman should eat that is good for recovery, lactation, and producing quality breast milk can be a hassle to families because rituals and food preferences differ in different provinces of China. What women should or should not eat can cause conflicts.

*My mother-in-law from Hunan would not cook “postpartum pig-feet soup” for me. But where I came from, this dish is considered most beneficial for postpartum women. “Pig-feet, egg, and ginger soup” or “pork bones soup” are to replenish and rebalance the”qi” and rid the body of “wind”. But in Hunan province, postpartum women are forbidden to have soup; they think it is not good for the production of breast milk. She also told me not to eat vegetables or fruits, as she believes that those will cause “wind” and “cold,” which will lead to tummy aches and flatulence for my breastfed baby, or that I will suffer from chronic headaches for years to come. I could not understand their eating style.* (P10 – first-time mother).

### Conflicts over infant care practices

While the baby is the center of the whole family, each family member has his / her own approach to caring for the baby. Some young mothers secretly wish that the grandparents would not insist on following outdated baby care practices. There can be disagreements between the parents and the grandparents of the child over their respective childcare practices.

*Putting too many clothes on the baby can be a problem leading to disagreements between me and my mother-in-law. I told her that she puts too many pieces of clothing on the baby, which could suffocate her, but my mother-in-law insisted. It is not cold here in Shenzhen, and one piece of clothing is enough, but my mother-in-law insists on wrapping up my baby with big blanket. I usually remove the blanket secretly when she is not looking.* (P22 – first-time mother).

*I do not feel at ease and cannot sleep well when my mother-in-law has the baby with her. I don’t know what she would do for the baby that I don’t agree with.* (P14 –second-time mother).

### Arguments in multigenerational families

The women appreciate the good intentions of their husband and members of their extended family in trying to help them and the newborn. However, the older generation’s “traditional” ways of caring for the baby may not be what women want. The women therefore sought to achieve mutual understanding of their needs and preferences from their husband and the older generation.

*I believe my mother-in-law has good intentions. But her traditional belief of what is best for me is not necessary what I want. It was not easy for me to take at the beginning. I now accept it, although I am not used to it.* (P19 – first-time mother).

*Both my mother and mother-in-law came to take care of me during the first month. My mother is from the village, but my mother-in-law has lived in the city for a long time. They could not get along. My mother-in-law insisted that I should not eat chicken, but my mother argued that it is best for me and my breast milk. I listened to my mother and ate chicken, then my husband pitched in and said that only rooster is acceptable. The three kept arguing over the issue. I felt exhausted, and finally stopped eating the chicken that my mother had brought from the village.* (P6 – second-time mother).

#### Preparing for the transition to parenthood / grandparenthood

The first-time mothers expressed a need to be better prepared for the newborn, while the second-time mothers had no such concern. The women believed that health professionals could help them to make a better transition to parenthood and achieve better intergenerational family relationships.

### Making arrangements before the arrival of the newborn

The new mothers believed that, apart from baby care, the antenatal classes should also have taught her and her husband some parenting skills to prepare them for their new role.

*I would have liked to learn more about baby care before the baby’s arrival. Hospitals should provide teaching and learning materials for expectant new parents. It would help new parents to be more assured about their new role.* (P5 – first-time mother).

### Offer husbands advice on how to support new mothers

The women also hoped that nurses would teach their husband skills on postpartum support and baby care. They feel that teaching their husband how to provide emotional support would be of help to them.

*I have worries during pregnancy and in the postpartum period, but my husband is not aware of it. It’s difficult for me to tell him of my feelings and unstable moods. It would be great if the nurses could tell him about my psychological changes, and what he can do to support me.* (P18 – second-time mother).

*My husband has not even asked me about my childbirth experience as if nothing had happened. He does not see that I really need his support and care. I would say that he does not know my sufferings and the impacts on me.* (P16 – first-time mother).

### Offer grandparents support in baby care

New mothers wished that nurses could offer grandparents advice on postpartum and baby care knowledge and skills to lessen their tension.

*I wish someone would tell my mother that her approach to baby care is outdated. While I don’t want to upset my mother, I wish she would do things that I am comfortable with. I hope the nurses can offer some new baby care skills to my mother.* (P3 – first-time mother).

*The old generation has their set of beliefs. They said that the newborn’s lanugo should be shaved, which I don’t agree with. They want to shave her eyebrows, too. I was shocked to hear that. My parents-in-law keep chasing after me for what they think is right. I have had to say no. Are there any nurses who would help me to explain this to them?* (P10 – first-time mother).

### Facilitate intergenerational communication

Many of the women wished that nurses would give advice or training to their mother / mother-in-law on postpartum and baby care. The women stated that their requests were not always understood by their family members and that they were consequently not given the support that they needed. They believed that advice from health professionals would carry some weight with their family members.

*My mother-in-law would do things her way and tell me this was in accordance with her experiences. It would be great if nurses would tell my mother-in-law that her approach to baby care is outdated …*. *I think it is necessary for “a third person,” especially a professional, to talk with the old generation.* (P10 – first-time mother).

### The need for coordinated and comprehensive postpartum home visit services

While most women welcomed the postpartum home visits, they suggested that it would be better to have a more comprehensive postpartum care program that catered to their needs. There were no differences in expectations regarding home visit services between the first and second-time mothers.

### Appreciative of the attention paid by home visit nurses to the psychological well-being of new mothers

The women appreciated the warm and continuous care shown by the home visit nurses, instead of “task-oriented” fragmented care.

*I really appreciate the “humanistic care” from home visit nurses. It is good to feel cared for*. (P3 – first-time mother).

*I think highly of the home visit nurses. They value the feelings of each new mother. For example, after I told them about my concerns when they visited, they sent WeChat messages to check up on me, and asked me if my concerns had been solved.* (P17 – first-time mother).

### Request for an online support program

While most of the women considered home visits by nurses to be helpful, they wanted to have access to advice whenever they needed it. These women suggested that internet-based or hotline phone support should be offered to allow for the timely receipt of information from health professionals.

*Sometimes I have queries about baby care that I need answers to right away. If there is a platform that allowed us to ask questions, I would be able to receive instant answers from a health professional and be reassured*. (P18 – second-time mother).

### Provide home tests for jaundice

The women also suggested that tests for jaundice be included in the home visits, so that they would not have to bring their baby to the clinic for an examination, because it is a Chinese cultural belief that women and babies should not go outdoors during the first postpartum month.

*It would be ideal if jaundice testing could be done at home so that I wouldn’t have to make a trip to the clinic. It is not advisable for me or the baby to go out in the first month. It is not easy for us to go outdoors, and I am afraid that we both will catch germs*. (P6 – second-time mother).

### Provide support for breastfeeding during home visits

Difficulty in breastfeeding was a common problem for the new mothers. The women requested that a breastfeeding consultant visit them at home. Women had sought breastfeeding consultations, since the service is not available in postpartum home visits.

*It would be great if a home visit nurse could offer breastfeeding consultations. I have galactostasis, and wish that the home visit nurse would be able to offer advice and help.* (P3 – first-time mother).

*I had a breastfeeding consultant visit me as a home visit nurse. She provided me with much knowledge and skills to enhance lactation, and also psychological counseling.* (P10 – first-time mother).

*One of my major concerns was my insufficient breast milk; I hope that health professionals can give me suggestions [on how to solve this problem].* (P14 – second-time mother).

## Discussion

This study explored the experiences and needs of Shenzhen women in the first 6 weeks after giving birth. The findings suggest that a woman, her husband, and mother / mother-in-law should be regarded as a whole unit when considering the postpartum needs of women in the Chinese culture. Support from a husband plays a crucial role in a woman’s postpartum recovery and in infant care [24, 25]. The significance of the provision of support to postpartum women from extended family members should also be noted. The study also identified room for improvement in the existing home visit services offered to postpartum women in Shenzhen.

Nine of the 22 women who took part in this study had delivered their babies by caesarean section (40.9%). Although this rate is consider high compared to the recommendation of World Health Organization [26], this is comparable to the national rate of China at 36.7% [27]. There was no indications that there were differences between these mothers with different mode of delivery in terms of their needs for postpartum care.

The first-time or second-time mothers in this study were cared for by their husband, mother, or mother-in-law, as well as by a “doing the month maid” during the postpartum period. In this study, the first-time mothers expressed more concerns about the changes in their body, while the second-time mothers were more worried about their physical recovery. All of the mothers considered nutritional supplements to be important for their recovery, and felt that their health would affect their baby’s health [28]. All of the mothers, regardless of whether they were first-time or second-time mothers, were uncertain about their infant care skills. They expressed worries about their baby’s feeding, elimination, and skin rashes, and about their ability to interpret their baby’s cries. This suggests that health professionals should provide new postpartum mothers with the knowledge and skills that they need to care for themselves [29, 30], as well as their newborn [24, 25].

Women required support from their husband and extended family [31, 32]. Both first-time and second-time mothers expressed the hope that their husband and extended family members could be better equipped with the knowledge and skills needed for postpartum and baby care [33, 34]. It was suggested that if mothers and grandmothers agreed on childcare practices, this would facilitate the transition of postpartum women into motherhood and ease the stress felt by postpartum women [33, 35].

Disagreements between a mother and her husband or mother / mother-in-law on postpartum and baby care practices could lead to emotional or psychological disturbances in postpartum women [36]. A study conducted in Shenzhen reported that postpartum women who lived with their parents-in-law were at a higher risk of exhibiting depressive symptoms than those who did not [37]. The involvement of a mother-in-law could cause a woman to struggle between respecting her mother-in-law and following her own beliefs on self-care and baby care practices [38]. The women in this study asked that their mother / mother-in-law be offered up-to-date knowledge and skills in baby care practices. Given that the psychological well-being of a new mother is affected by the support that she receives from her husband or extended family members [38, 39], it should be beneficial to facilitate intergenerational communication between new parents and grandmothers, thereby enhancing mutual understanding in postnatal care and baby care and improving family relationships [38].

The women in this study expressed the hope that health professionals would help them to better prepare for the transition to motherhood. They also emphasized that there is a need to prepare the whole family to assume new roles, such as that of father and grandparents. Since in China, it is a family practice and most grandparents help their adult children to provide care for their grandchildren [40]. In this study, 20/22 (90.9%) women were cared by their mother or mother-in-law. Therefore, we proposed that a family-based intervention should be applicable and recommended for Chinese families. Hence, with the understanding of the postpartum woman that family members should be involved, support on postpartum and baby care should be given to the whole extended family [33].

Unlike studies on postpartum home visits in other parts of China [12, 13], the Shenzhen women in this study held positive attitudes towards the postpartum home visit services that they received. They also suggested that women be provided with advice on breastfeeding issues or nipple soreness, and that jaundice testing for babies be offered at home. Another suggestion was that timely consultations be offered to queries on self-care or baby care practices [41, 42]. Online consultations should be developed to offer women in Shenzhen more comprehensive postnatal care services.

### Strengths and limitations

This is the first qualitative study to assess the needs of early postpartum women in Shenzhen. This study provided an in-depth understanding of the concerns expressed by the women and their service needs. The findings from this study also shed light on how to enhance existing postpartum home visit services.

This study is limited in that only postpartum women were included in the interviews. The concerns of the women regarding family support were not confirmed by their husband or extended family members. The experiences of other family members as caregivers were not explored. A further study is needed to explore family relationships and dynamics, and concerns about intergenerational involvement in postpartum and child care.

## Conclusion

This qualitative study explored the experience and health needs of postpartum women and identified the gaps in existing postpartum home visit services. The women’s concerns during the postpartum period were related to their need to recover physically and to their perceptions about their proficiency in caring for their infant. Support from husbands and grandmothers could facilitate or impede the women’s transition to motherhood, and there were disagreements / conflicts over intergenerational beliefs regarding postpartum and child care.

Quality postpartum care should also take into account the expectations and concerns of women about family support and intergenerational conflicts over child care [25, 43]. In providing postpartum care to women with the involvement of extended family members, health professionals should consider the family as a whole, and tailor services to the needs of the whole family, taking into account family structure and the interactions between family members of different generations in relation to postpartum and baby care.

## Supplementary information

**Additional file 1.** Demographic information sheet

**Additional file 2.** Interview guide

## Data Availability

All data generated or analyzed during this study are included in this manuscript.
