# Supplementary material for: The experiences of early postpartum Shenzhen mothers and their need for home visit services: a qualitative exploratory study
Source: BMC Pregnancy Childbirth. 2019 Dec 31;20:5. doi: 10.1186/s12884-019-2686-8 (PMC6938610; doi:10.1186/s12884-019-2686-8)
Supplement: Supplementary file 1 — Additional file 1. Demographic information sheet [file 12884_2019_2686_MOESM1_ESM.docx]

Questionnaire for demographic information

Case NO: Date of assessment:

1. Mother’s information:
   1. Age ______________________________
   2. Parity : 1 □ 2 □
   3. Mode of delivery:

Vaginal delivery □ Vaginal assisted delivery □

Elective cesarean section □ Emergency cesarean section □

- 1. Day after childbirth:_________________
  2. Length of Maternity Leave: _________________
  3. Who care for you during the postpartum period:

Husband □ Your own mother □ /father □ Mother □ /Father in law □ Doing the month maid □ Other relatives □ Nobody □

- 1. Home town of your caregiver? _____________Province City □ Village □
  2. Family Income per month :

5000RMB or lower □ 5001-10000RMB □ 10001-20000RMB □ 200001-30000 RMB □ 30001RMB or above □

- 1. Infant Gender: Boy □ Girl □

(11) Mode of baby feeding: Exclusive breastfeeding □ Baby formula □ Mixed□
