# Supplementary material for: The experiences of early postpartum Shenzhen mothers and their need for home visit services: a qualitative exploratory study
Source: BMC Pregnancy Childbirth. 2019 Dec 31;20:5. doi: 10.1186/s12884-019-2686-8 (PMC6938610; doi:10.1186/s12884-019-2686-8)
Supplement: Supplementary file 2 — Additional file 2. Interview guide [file 12884_2019_2686_MOESM2_ESM.docx]

Interview guide for postpartum women

1. Can you tell me your experience during your postpartum period?
2. How do you feel after being a new mother? Have you encountered any discomfort?
3. What are the things that you are concerned about?
4. Has your husband been involved in baby care? Do you think his help is adequate?
5. Do you think the help from the caregiver is enough? How do you think of their help?
6. How do you think the home visit service? What other services do you would like to receive from us?
7. Is there anything you think we could do to help you and your husband? And your family?
8. Is there anything else you would like to tell me?
